# Collecting Symptoms and Sensor Data With Consumer Smartwatches (the Knee OsteoArthritis, Linking Activity and Pain Study): Protocol for a Longitudinal, Observational Feasibility Study

**DOI:** 10.2196/10238

**Published:** 2019-01-23

**Authors:** Anna L Beukenhorst, Matthew J Parkes, Louise Cook, Rebecca Barnard, Sabine N van der Veer, Max A Little, Kelly Howells, Caroline Sanders, Jamie C Sergeant, Terence W O'Neill, John McBeth, William G Dixon

**Affiliations:** 1 Arthritis Research United Kingdom Centre for Epidemiology Centre for Musculoskeletal Research University of Manchester, Manchester Academic Health Science Centre Manchester United Kingdom; 2 National Institute for Health Research Manchester Musculoskeletal Biomedical Research Centre Manchester University National Health Service Foundation Trust Manchester Academic Health Science Centre Manchester United Kingdom; 3 Centre for Health Informatics Division of Informatics, Imaging and Data Sciences University of Manchester Manchester United Kingdom; 4 Health eResearch Centre The United Kingdom Farr Institute of Health Informatics Research Manchester United Kingdom; 5 Mathematics Group Aston University Birmingham United Kingdom; 6 Human Dynamics Group Massachusetts Institute of Technology Media Lab Massachusetts Institute of Technology Cambridge, MA United States; 7 Nuffield Department of Clinical Neurosciences University of Oxford Oxford United Kingdom; 8 The National Institute for Health Research School for Primary Care Research Manchester Academic Health Science Centre Manchester United Kingdom; 9 Centre for Primary Care Faculty of Life Sciences University of Manchester Manchester United Kingdom; 10 National Institute for Health Research Greater Manchester Patient Safety Translational Research Centre University of Manchester Manchester United Kingdom; 11 Centre for Biostatistics University of Manchester, Manchester Academic Health Science Centre Manchester United Kingdom; 12 Department of Rheumatology Salford Royal National Health Service Foundation Trust Salford United Kingdom

**Keywords:** medical informatics computing, mHealth, patient-reported outcomes, musculoskeletal diseases, mobile phone

## Abstract

**Background:**

The Knee OsteoArthritis, Linking Activity and Pain (KOALAP) study is the first to test the feasibility of using consumer-grade cellular smartwatches for health care research.

**Objective:**

The overall aim was to investigate the feasibility of using consumer-grade cellular smartwatches as a novel tool to capture data on pain (multiple times a day) and physical activity (continuously) in patients with knee osteoarthritis. Additionally, KOALAP aimed to investigate smartwatch sensor data quality and assess whether engagement, acceptability, and user experience are sufficient for future large-scale observational and interventional studies.

**Methods:**

A total of 26 participants with self-diagnosed knee osteoarthritis were recruited in September 2017. All participants were aged 50 years or over and either lived in or were willing to travel to the Greater Manchester area. Participants received a smartwatch (Huawei Watch 2) with a bespoke app that collected patient-reported outcomes via questionnaires and continuous watch sensor data. All data were collected daily for 90 days. Additional data were collected through interviews (at baseline and follow-up) and baseline and end-of-study questionnaires. This study underwent full review by the University of Manchester Research Ethics Committee (#0165) and University Information Governance (#IGRR000060). For qualitative data analysis, a system-level security policy was developed in collaboration with the University Information Governance Office. Additionally, the project underwent an internal review process at Google, including separate reviews of accessibility, product engineering, privacy, security, legal, and protection regulation compliance.

**Results:**

Participants were recruited in September 2017. Data collection via the watches was completed in January 2018. Collection of qualitative data through patient interviews is still ongoing. Data analysis will commence when all data are collected; results are expected in 2019.

**Conclusions:**

KOALAP is the first health study to use consumer cellular smartwatches to collect self-reported symptoms alongside sensor data for musculoskeletal disorders. The results of this study will be used to inform the design of future mobile health studies. Results for feasibility and participant motivations will inform future researchers whether or under which conditions cellular smartwatches are a useful tool to collect patient-reported outcomes alongside passively measured patient behavior. The exploration of associations between self-reported symptoms at different moments will contribute to our understanding of whether it may be valuable to collect symptom data more frequently. Sensor data–quality measurements will indicate whether cellular smartwatch usage is feasible for obtaining sensor data. Methods for data-quality assessment and data-processing methods may be reusable, although generalizability to other clinical areas should be further investigated.

**International Registered Report Identifier (IRRID):**

DERR1-10.2196/10238

## Introduction

The increasing uptake of consumer wearable devices provides an opportunity for health data collection in people’s natural environments. Wearable devices permit frequent collection of patient-reported outcomes via touchscreen questionnaires alongside passively collected measures of behavior via sensors (eg, physical activity). This may help develop novel insights into conditions with symptoms that are otherwise difficult to track. Osteoarthritis is an example of such a condition. It is a prevalent, degenerative condition [1,2] where fluctuating pain and loss of mobility are the major symptoms. In knee osteoarthritis, increased physical activity may exacerbate knee pain. Conversely, certain forms of exercise are known to have a beneficial effect on pain symptoms [3,4]. Characterizing the relationship between pain and activity could help in the development of targeted interventions. However, in the past, it has been challenging to capture self-reported pain symptoms alongside objective measurements of physical activity. Typically, patients are asked to summarize or recall pain over large time periods (eg, “in the last week” or “generally this month”) in paper-based questionnaires and self-report activity. Having continuous activity data alongside frequent pain reports would improve data quality and reduce recall bias.

Wearable consumer devices are increasingly popular as fitness tools [5]. Recently, consumer-grade cellular smartwatches (eg, Apple Watch, Huawei Sawshank, and LG Urbane) have been introduced to the market. These watches have similar functionalities as a mobile phone. Users can use them to make phone calls, navigate using global positioning system (GPS), or check emails. Like mobile phones, they have full-color touch screens, and like activity trackers (eg, Fitbit), they have a wide range of sensors that can measure users’ behavior. Smartwatches could potentially be used to capture health-related data for research or clinical practice. Although the devices and accompanying software are well developed, various questions remain unanswered. Would participants wear the devices and self-report outcomes for a longer period of time? How can missing sensor data be handled? Is sensor data quality from smartwatch sensors sufficient? How can researchers or clinicians convert high volumes of sample-rate sensor data to meaningful outcomes? These questions need to be answered before consumer wearables can be used as novel interventions or to improve outcome assessment in clinical trials.

**Figure 1:**
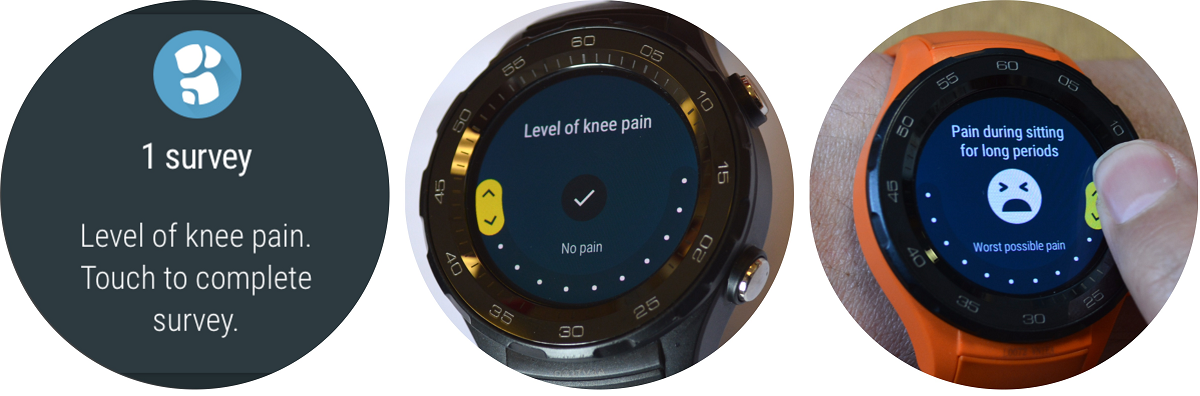
Images of the Knee OsteoArthritis, Linking Activity and Pain app user interface; left: notification of an active survey and start screen of questionnaire; middle: data entry screen for survey “level of knee pain”; right: data are entered by swiping the numeric rating scale icon.

In this study, we developed a smartwatch app to collect patient-reported outcomes alongside sensor data using an Android Wear cellular smartwatch (Figure 1). The app was developed in collaboration with the Google Fit & Android Wear groups at Google UK.

The overall aim of this study was to investigate the feasibility of using consumer smartwatches as a novel tool to capture data on pain (multiple times a day) and activity (continuously) for 3 months in patients with knee osteoarthritis.

Specific study objectives were to test the feasibility, acceptability, and ongoing engagement with smartwatch data collection for research; to explore motivations, health behavior, and perceived impact of sensor data collection and frequent symptom reporting; to examine the association between twice-daily symptoms and weekly/monthly validated osteoarthritis questionnaires; and to explore the relationship between self-reported pain and activity levels. In addition, the analysis of exploratory observational data gathered in this study may serve as the first step toward the development of new outcome measures for remote monitoring of disease severity for use in clinical practice and research that incorporate both physical activity and pain.

## Methods

### Overview

For this study, data were collected in three ways: via consumer cellular smartwatches (all participants), participant interviews at the beginning and end of the study (subset of participants), and via a baseline and end-of-study questionnaire (all participants). In this section, we first provide an overview of participants and the study process and then specify the data-collection methods.

### Study Design

#### Participants and Recruitment

Eligibility criteria for participants were the presence of knee osteoarthritis (self-reported), age of 50 years or above, living in the Greater Manchester area or willing to travel to Manchester, owning a smartphone, and willing to participate in the *Cloudy with a Chance of Pain* study.

In July 2017, the study was advertised in local newspapers and magazines and via social media channels. Interested participants were invited to contact the study team, after which they were sent a patient information sheet and invitation to one of four enrollment events in September 2017.

#### Study Duration

The study was designed as a feasibility study in which people with knee osteoarthritis were asked to wear a consumer cellular smartwatch for 90 days. Additionally, participants were invited for voluntary participation in interviews at baseline and after completion of the study. The study was nested within an existing mobile phone study—*Cloudy with a Chance of Pain*—that examined the relationship between weather and pain among people with long-term pain conditions [6,7].

#### Smartwatches

The Huawei Watch 2 was used for the study. Google UK provided these cellular smartwatches with subscriber-identity module cards (enabling data collection and direct transmission, independent from a mobile phone). The watches were preinstalled with the Knee OsteoArthritis, Linking Activity and Pain (KOALAP) app developed by the Google Android Wear team in collaboration with the researchers (Figure 1). This app passively collected raw sensor data and launched various questionnaires to collect patient-reported outcomes (see Data Collection section). Patients wore the watch on the wrist that they found most comfortable.

#### Enrolment Event

At the enrolment event, participants provided written consent, completed the baseline questionnaire, and received the study smartwatch and user guide. Participants were asked whether they were willing to be considered as potential participants for two additional interviews (one at baseline and one at follow-up). Staff from Google attended the enrolment events to address any technical questions that arose. After setting up the smartwatch, participants downloaded the *Cloudy with a Chance of Pain* app on their mobile phone. During the setup of their *Cloudy* account, participants entered their unique KOALAP identifier for later pairing of data from both sources.

#### Study End

Participants returned their smartwatch in January 2018 (during the follow-up interview or by prepaid postage). They were each sent a link to an electronic end-of-study questionnaire about their experiences with the watch. Participants were given a £10 shopping voucher for completing the feedback questionnaire and for each interview they participated in, and they were reimbursed reasonable travel costs.

### Data Collection

#### Smartwatch Use

Participants were asked to wear the smartwatch shortly after waking until going to bed. They were asked to respond to all symptom questionnaire notifications they received via the watch (maximum of 6 on Sundays, 5 on Wednesdays, and 4 on other days). Participants charged their smartwatches overnight. During charging of the watch, participants’ activity and questionnaire data were uploaded to the servers (see Data Storage and Transfer section). Self-reported symptom data and passively collected sensor data were collected from participants via the smartwatch.

#### Self-Reported Symptom Data

During the smartwatch setup on Day 1, participants answered questions A1 to A4 displayed in Table 1. The answers to question A2 to A4 were used in the recurring watch questions during the main study.

**Figure 2:**
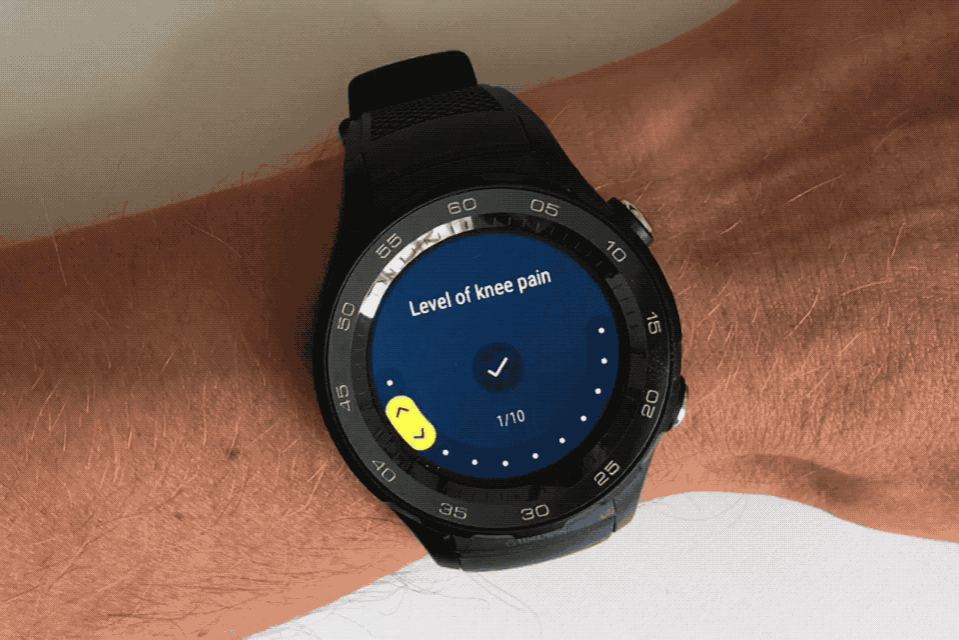
Example of the input screen for the numerical rating scale in the Knee OsteoArthritis, Linking Activity and Pain app.

Four to five times a day, the watch app activated questions B1 to B5 (Table 2; Figure 2). These questions asked patients to record on 0-10 numeric rating scales the level of knee pain (twice daily), to what extent the knee pain affected their daily activities (daily), the level of knee pain after the important activity specified upon enrolment (daily), to what extent knee pain had prevented them from doing their painful activity specified upon enrolment (weekly), and their quality of life (weekly). An animated version of the user interface for answering questions is available in Multimedia Appendix 1.

In addition to the daily and weekly questions B1 to B5 (Table 2), participants were asked to answer 26 questions on their pain and function (monthly, on Days 14, 44, and 74 from the start point). These were taken from the standard Knee injury and Osteoarthritis Outcome Score (KOOS) questionnaire [8] (pain domain: Q1 to Q9, activities of daily life domain: Q1 to Q17) and rated on a 5-point Likert scale. We used only two KOOS subscales from the full KOOS questionnaire (42 items) to reduce the burden of data entry for participants.

Participants were alerted to the twice-daily and daily questions with watch buzzer vibrations. Questions opened on touching the notification. The watch vibrated when the survey was triggered and, if the questionnaire was not answered, every 2 hours until expiry of the question window. This time window comprised 4 hours for the twice-daily questionnaire, 7 hours for the daily questionnaire, 12 hours for the weekly questionnaire, and 7 days for the monthly questionnaire (Table 2). If participants did not answer a questionnaire within a fixed time period, the questionnaire was automatically dismissed. The watch did not vibrate after 9 PM. To avoid alert fatigue, the weekly and monthly questionnaires did not generate additional vibrations.

#### Sensor Data

**Figure 3:**
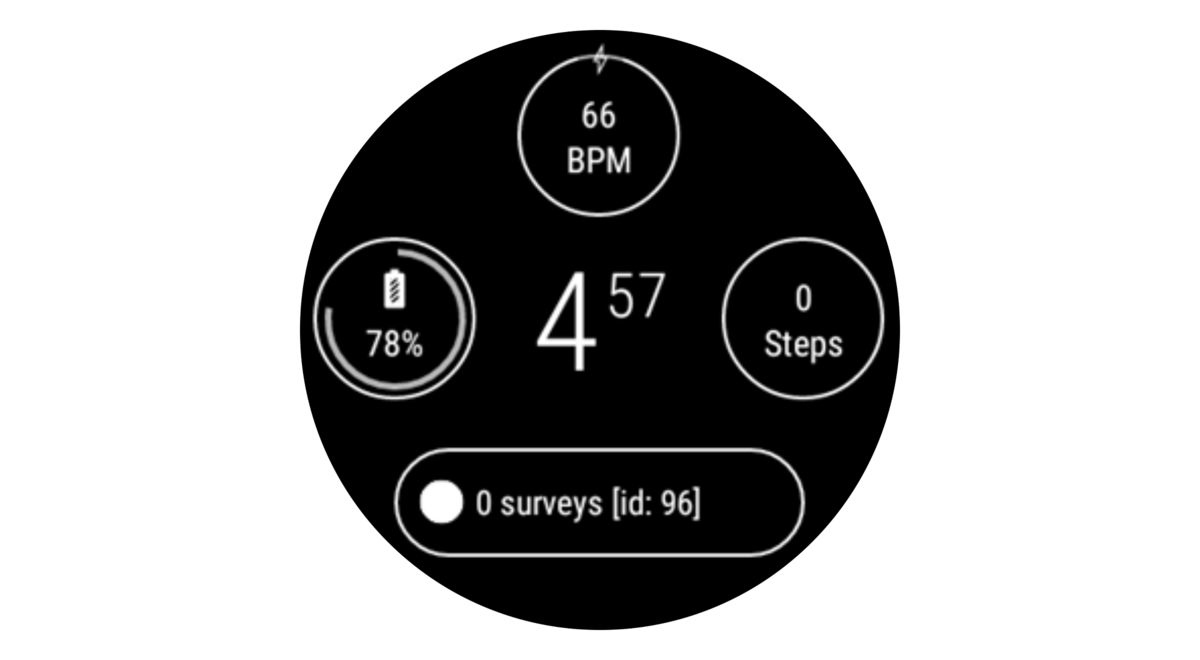
Watch homescreen of the Knee OsteoArthritis, Linking Activity and Pain app.

The KOALAP app collected sensor data on the inertial measurement unit (accelerometer, gyroscope, and magnetometer) at 50 Hz, estimated pulse rate at 1 Hz, and barometer once per minute. These sampling frequencies balanced the battery life and data-collection frequency. Although the smartwatch was capable of collecting GPS data, this function was not used in order to achieve a battery life of 10-12 hours. To further preserve battery life, all smartwatch apps apart from the study app were disabled, and the watch was permanently prevented from data transmission (in “airplane mode”) until docked to a charger at night. Apart from the study app, participants could see a home screen that included the time, their daily step count, their last-measured pulse rate (with an option to see data from the complete day), and battery status. Figure 3 shows the home screen at 4:57 AM for a participant who has taken 0 steps, a heart rate of 66 beats per minute, a remaining battery life of 78%, and no outstanding questionnaires (or surveys) to complete.

**Table 1:** Baseline data items.

Item	Questions	Multiple choice answers
A1	In which of the following sites do you have OA^a^? (max 5)	Hand(s), Shoulder(s), Hip(s), Ankle(s), Foot/ feet
A2	In which knee is your OA typically more troublesome? (max 1)	Left, Right
A3	Thinking about your (A2: right/left) knee, what is the one activity, or action, that consistently causes you the most knee *pain*? (max 1)	Standing, Walking, Turning/twisting, Sitting for long periods, Sitting to standing, Squatting/bending/kneeling, Walking up stairs/inclines
A4	Thinking about your (A2: right/left) knee, what is the one activity, or action, most *important* for you to be able to do with minimal pain and difficulty? (max 1)	Socialise, Walk, Play sport, Do household tasks, Work effectively, Get washed and dressed

^a^OA: osteoarthritis.

**Table 2:** Questionnaire timings—vibrating notification trigger and completion window times.

Item	Frequency	Trigger time	Window	Question
B1	Twice daily	12:22 PM and 6:22 PM	12:22 PM-4 PM and 6:22 PM-10 PM	Level of knee pain
B2	Daily	5 PM	5 PM-12 AM	Knee pain affecting daily activities
B3	Daily	5 PM	5 PM-12 AM	Knee pain after (important activity A4)
B4	Weekly	Wednesday 12 PM	Wednesday 12 PM-12 AM	Knee pain preventing (painful activity A3)
B5	Weekly	Sunday 12 PM	Sunday 12 PM-12 AM	Quality of life
KOOS^a^	Monthly	Days 14, 44, 74 from start point	1 week	26 questions from KOOS questionnaire

^a^KOOS: Knee injury and Osteoarthritis Outcome Score.

#### Data Collection via Mobile Phone

Via the *Cloudy with a Chance of Pain* mobile phone app, participants received a notification every day (default time 6:24 PM) to rate 10 aspects of their symptoms in the app on a five-point ordinal scale [6,7]. Optionally, participants could answer (any of) the 10 aspects additional times in a day, for example, in the case of changing pain. In parallel, the mobile phone’s location services passively recorded geolocation hourly to enable collection of local weather data.

#### Data Collection via Participant Interviews

Participant interviews were conducted to explore motivations, health behavior, and perceived impact of activity monitoring for self-management and health behavior. All participants were invited to participate in two interviews (at baseline and end of study), and 19 participants (73%) agreed to participate. Sixty-minute interviews were conducted by a university researcher (KH) at the on-boarding/off-boarding events or the participant’s home. Interviews were semistructured and guided by an interview schedule. Participants were asked about their general health and their experiences of living with osteoarthritis. Their motivations, expectations of the study, and previous use of electronic Health (eHealth) technologies were also explored. The interviews were analyzed using a grounded theory approach. Transcripts were coded using NVIVO by the research team, who met monthly to discuss emerging themes. Audio recordings from the interviews will be archived for a period of 10 years. Google will be provided with a copy of the summary report analysis of interview and questionnaire responses, but will not have access to interview audio recordings or transcripts.

#### Sample Size

A minimum sample size of 20 participants was required based on expected attrition. In a previous study assessing feasibility and acceptability of data collection via mobile phones in a population with arthritis, 30% of participants withdrew from the study [6].

#### Data Storage and Transfer

Smartwatch data were stored temporarily on the smartwatch in its SQLite database. When participants charged the watch, the watch stopped collecting data, disabled the airplane mode, uploaded all data to the server over 4G, and erased data from the watch. If the internal memory of the smartwatch was full, the watch stopped collecting data until it was charged again, and data were then successfully uploaded to the servers. This only happened if data were not uploaded to the servers for several days, because 4G connectivity was poor at the location of charging or because participants were abroad (no 4G connectivity).

The anonymized data were transferred in encrypted form over HTTPS to a remote server hosted by Google, where they were stored encrypted at rest in Spanner (Google LLC), Google’s globally distributed NewSQL database. At no point were the data linked or will be linked to personally identifiable information such as name or email address. Details of Google’s data center security are provided [9].

The decryption key to participants’ anonymized data was stored securely on two separate university servers. At no time was the key shared with Google. Google will not have access to participants’ names and will not therefore be able to personally identify any study participant. Google will only access the data for quality-control purposes and will not use the data for any other purpose. At the end of the study, once the university research team has indicated it is satisfied that all data have been received, Google will delete the data collected and provide written confirmation of data destruction.

### Analysis

In this section, we present the analysis methods per the study objectives described in the Introduction.

#### Feasibility, Acceptability, and Ongoing Engagement

To assess feasibility, we will examine data completeness. For the sensor data, we will examine whether actual sampling frequencies are at least as high as that specified during app design. For the questionnaire data, we will examine the percentage of questions answered per day and per participant per day. To assess acceptability, answers to the relevant questions of the end-of-study questionnaire will be summarized as a percentage of participants selecting a multiple-choice option/giving a similar open answer. Patterns of engagement through time will be described with descriptive statistics per participant, such as percentage of questions answered (per day or per participant per day), hours of wearing the watch, and time in study.

#### Motivations, Perceived Impact of Continuous Passive Monitoring, Symptom Reporting, and Health Behavior

Thematic analysis (drawing on techniques of a grounded theory approach) will be used to identify initial themes and explore relations between themes and across cases (using constant comparison). In addition, relevant questions from the end-of-study questionnaire will be summarized as a percentage of participants selecting a multiple-choice option/giving a similar open answer.

#### Association Between Twice-Daily Symptoms and Weekly and Monthly Symptoms

We will examine the association between twice-daily and weekly symptom reports, including the variability in the twice-daily responses within the week. This analysis will have an exploratory nature and focus on generating hypotheses for future research. Panel linear regression and latent growth models will be used to assess how pain varies over the repeated observations (as reported in up to 4680 twice-daily questions, 2340 of each of the daily questions, 364 weekly questions, and 78 monthly surveys of 17 KOOS questions). Further exploratory work may investigate whether the variation in pain is homogenous throughout the sample (eg, with multilevel models) or whether some factors moderate/mediate these trajectories.

#### Relationship Between Self-Reported Pain and Activity Levels

Significant sensor data signal processing will be required to translate the raw sensor output into clinically meaningful variables. The physical activity outcomes we aim to create from the sensor data include amount of physical activity, characteristics of painful walking, and activity patterns that may aggravate pain.

Approaches to examine the relationship between symptom data and sensor data will likely include several processing steps such as extracting gravitational orientation vectors, computing dynamic body acceleration vectors, extracting properties of these vectors such as magnitude and direction, segmenting magnitude and direction vectors into behaviorally contiguous time regions, extracting a range of features from these regions, and identifying regions that are most likely to correspond to gait or other behaviors implicated in pain aggravation. For these regions, measures of patterns of behavior relevant to patients with osteoarthritis can be estimated (eg, step count, time spent in sedentary behaviors, and time spent in motorized or other transport activities). These measures will then be compared to the self-reported measures using appropriate techniques (eg, prediction errors for interval self-report scales or classification errors for nominal scales). Self-reported scales will be interpolated to make such comparisons against continuous sensor data measures meaningful. Based on the processing of sensor data described above, we will explore patterns of physical activity-related behavior in participants. The metrics of physical activity derived from the sensor data will be summarized for all participants.

## Results

Here, we specify the user interface of the KOALAP smartwatch application, the timelines for the study, and the review processes the study has undergone.

### User Interface

Figures 1-3 show the user interface of the KOALAP smartwatch app. Multimedia Appendix 1 presents an animated version of Figure 2 that shows how data are entered in the user interface.

### Timelines

Participants have been recruited in September 2017. Data collection via the watches was completed in January 2018. Collection of qualitative data through patient interviews is still ongoing. Data analysis will commence when all data are collected; results are expected in 2019.

### Ethics

This study underwent full review by the University of Manchester Research Ethics Committee (#0165) and University Information Governance (#IGRR000060). For the qualitative data analysis, a system-level security policy was developed in collaboration with the University Information Governance Office. The project also underwent an internal review process at Google, including separate reviews of accessibility, product engineering, privacy, security, legal, and protection regulation compliance.

The results from this study will be disseminated at national and international conferences as well as in peer-reviewed journals and, where possible and appropriate, at public engagement events.

## Discussion

KOALAP is the first health study to use consumer cellular smartwatches to collect self-reported symptoms alongside sensor data for musculoskeletal disorders. This feasibility study will assess the practicalities of recruitment and acceptability of using smartwatches to collect symptom and sensor data. In addition, the study will examine the relationship between passively recorded physical activity and patient-reported knee osteoarthritis symptom reports.

Although statistical power will be limited in this feasibility study, it will be the first step toward new methods for collecting health data and possibly generating novel outcomes.

The results of the feasibility study will be used to inform the design of future mobile health studies. Results for the first two objectives (feasibility and participant motivations) will inform future researchers whether or under which conditions cellular smartwatches are a useful tool to collect patient-reported outcomes alongside passively measured patient behavior. The third objective (exploration of associations between self-reported symptoms at different moments) will contribute to our understanding of whether it may be valuable to collect symptom data more frequently. Sensor data–quality measurements will indicate whether cellular smartwatch usage is feasible for obtaining sensor data. Methods for data-quality assessment and data-processing methods may be reusable, although generalizability to other clinical areas should be further investigated.

## Appendix

Multimedia Appendix 1 Animated version of the symptom-input screen of the Knee OsteoArthritis, Linking Activity and Pain App with the numerical rating scale.

Multimedia Appendix 2 Electronic feedback questionnaire.

Multimedia Appendix 3 Peer-reviewer report from Arthritis Research UK.

## Abbreviations

eHealth: electronic Health
GPS: global positioning system
KOALAP: Knee OsteoArthritis, Linking Activity and Pain
OA: osteoarthritis
KOOS: Knee injury and Osteoarthritis Outcome Score
