# Supplementary material for: Collecting Symptoms and Sensor Data With Consumer Smartwatches (the Knee OsteoArthritis, Linking Activity and Pain Study): Protocol for a Longitudinal, Observational Feasibility Study
Source: JMIR Res Protoc. 2019 Jan 23;8(1):e10238. doi: 10.2196/10238 (PMC6366393; doi:10.2196/10238)
Supplement: Multimedia Appendix 2 [file resprot_v8i1e10238_app2.pdf]

Knee Osteoarthritis: Linking Activity and Pain (Koalap)

A sub-study within Cloudy with a Chance of Pain

Self-administered survey for all participants

Motivation

1. What motivated you to participate in this study? **[Free text]**

Sensor/Watch/Hardware

2. How frequently did you wear the watch/sensor during the study?

Always Often Sometimes Rarely Never

3. If you did take the watch off, when was this? Please circle all that apply

When charging it, **Whilst showering, In the bath, When swimming, In an exercise class, At the gym, Whilst cooking, Socialising, When at work, At night in bed, Other – please specify**

4. The watch/sensor stopped me from going about my normal activities during the day.

Strongly agree Agree Neither agree nor disagree Disagree Strongly disagree

5a. I found the watch/sensor to be comfortable to wear.

Strongly agree Agree Neither agree nor disagree Disagree Strongly disagree

5b. I often forgot to charge the watch or wear it again after charging

Strongly agree Agree Neither agree nor disagree Disagree Strongly disagree

Manual data entry

6. Did you fill out the surveys about your OA pain levels on your watch **on the day** when you were prompted?

Always Often Sometimes Rarely Never

7. I found the timing of the lunchtime prompt to fill out the daily pain logging surveys to be convenient for me.

Strongly agree Agree Neither agree nor disagree Disagree Strongly disagree

8. I found the timing of the evening prompt to fill out the daily pain logging surveys to be convenient for me.

Strongly agree Agree Neither agree nor disagree Disagree Strongly disagree

9. I found the timing of the evening prompt meant I was unable to record important things that happened that evening.
Strongly agree Agree Neither agree nor disagree Disagree Strongly disagree

10. I found the timing of the prompt to fill out the weekly pain logging surveys to be convenient for me.

Strongly agree Agree Neither agree nor disagree Disagree Strongly disagree

11. I found the timing of the prompt to fill out the monthly pain logging surveys to be convenient for me.

Strongly agree Agree Neither agree nor disagree Disagree Strongly disagree

12. I found the prompts to fill out the pain logging surveys to be disruptive.

Strongly agree Agree Neither agree nor disagree Disagree Strongly disagree

13a. I thought the frequency of the pain logging survey prompts was too high.

Strongly agree Agree Neither agree nor disagree Disagree Strongly disagree

13b. I thought the frequency of the pain logging survey prompts was too low.

Strongly agree Agree Neither agree nor disagree Disagree Strongly disagree

13c. *Where* and at *what time* did you normally fill out the pain logging surveys?

[open ended answer]

14. I found the format and wording of the OA pain level surveys easy to understand

Strongly agree Agree Neither agree nor disagree Disagree Strongly disagree

15. I found it easy to enter my manual pain level data into the watch app

Strongly agree Agree Neither agree nor disagree Disagree Strongly disagree

<u>Presentation of Activity Data</u>

16. I checked my activity data on the watch…

More than once a day Daily Every few days Less than once a week Never

17. I found the way my activity data was presented was easy to understand

Strongly agree Agree Neither agree nor disagree Disagree Strongly disagree

18. I enjoyed being able to view my activity data on the watch

Strongly agree Agree Neither agree nor disagree Disagree Strongly disagree

19. How would you improve the presentation of the physical activity data? **[free text]**

20. I checked my heart rate data on the watch…

More than once a day Daily Every few days Less than once a week Never

21. I found the way my heart rate data was presented was easy to understand

Strongly agree Agree Neither agree nor disagree Disagree Strongly disagree

22. I enjoyed being able to view my heart rate data on the watch

Strongly agree Agree Neither agree nor disagree Disagree Strongly disagree

<u>Other</u>

23. I would participate in a similar study again if I had the opportunity.

Strongly agree Agree Neither agree nor disagree Disagree Strongly disagree

24. Is there anything that could have made your participation in this study more useful or easy for you? **[free text]**

25. Are there any changes you would suggesting making to the watch? **[free text]**
