# Supplementary material for: Collecting Symptoms and Sensor Data With Consumer Smartwatches (the Knee OsteoArthritis, Linking Activity and Pain Study): Protocol for a Longitudinal, Observational Feasibility Study
Source: JMIR Res Protoc. 2019 Jan 23;8(1):e10238. doi: 10.2196/10238 (PMC6366393; doi:10.2196/10238)
Supplement: Multimedia Appendix 3 [file resprot_v8i1e10238_app3.pdf]

Internal (Arthritis Research UK follow up)

- There are still some concerns about the recruitment plan and how well the BBC programme will recruit participants to the study. Is there a contingency plan if the BBC pull out?
- If you put an app on someone's iPhone, Apple gives you access to data on other apps people have on their phone and that can give you intel on the interests of your audience. Would it be possible for Arthritis Research UK to get a report from Will on this? So it's not personal data but it would help us to build up more detailed personas on our audiences?
- The Brand and Digital teams would like a hands on version of the app as soon as possible (once an award is made).
- Do you have a timescale of when you will require Arthritis Research UK input/support?

Reviewer 1

This is a very innovative project. The methods/approach is appropriate as is the analysis plan. The team is outstanding. Dissemination plan is solid.

My only concern would be to ensure that the participants in the study give their permission for the data to be used for the citizen science experiment.

Reviewer 2

This is an engaging project and ticks several key areas of interest for the charity - proms, use of mobile apps for monitoring symptoms/ disease activity and increasing patient engagement and agency .

I can also see that this would be good publicity for the charity.

Generally, I thought this was a good proposal which was fairly clear and thought out. I have a couple of concerns, firstly the self-selecting nature of patients and reporting of symptoms. How will the stratification into cohorts such as R.A/ O.A/ primary pain etc. work with a self-reporting group of patients? For example, are your R.A. cohort going to have to declare an existing diagnosis?

Secondly and this is one that comes up with all pain discussions, pain experienced may not be due to MSK conditions but may have other causes on particular days/ weeks. Does this matter? I can see that data on pain and meteorological conditions could still be useful but more limited for MSK sufferers.

Thirdly, I am a fan of citizen science projects but the big data ones do have a large dropout rate in terms of participation. It is asking a lot to report daily for 6 months and I would anticipate a high drop out. That said, the pilot study is promising and they suggest some ways of maintaining ongoing engagement.

As long as the media deadlines and publicity don't interfere with the quality of the research, it seems like there is potential here which could have a number of interesting offshoots.

Reviewer 3

I am broadly supportive of the charity funding the project with a few caveats:

- Will specific diagnoses be input prior to any analysis?

- Where will the copyright of the app and any profit fall?
